# Snap-shots of cluster growth: structure and properties of a Zintl ion with an Fe_3_ core, [Fe_3_Sn_18_]^4−^†

**DOI:** 10.1039/d3sc04709a

**Published:** 2023-12-02

**Authors:** Zi-Sheng Li, Wei-Xing Chen, Harry W. T. Morgan, Cong-Cong Shu, John E. McGrady, Zhong-Ming Sun

**Affiliations:** a Department of Chemistry, University of Oxford South Parks Road Oxford OX1 3QR UK john.mcgrady@chem.ox.ac.uk; b State Key Laboratory of Elemento-Organic Chemistry, Tianjin Key Lab for Rare Earth Materials and Applications, School of Materials Science and Engineering, Nankai University Tianjin 300350 China sunlab@nankai.edu.cn

## Abstract

The endohedral Zintl-ion cluster [Fe_3_Sn_18_]^4−^ contains a linear Fe_3_ core with short Fe–Fe bond lengths of 2.4300(9) Å. The ground state is a septet, with significant σ and π contributions to the Fe–Fe bonds. The Sn_18_ cage is made up of two partially fused Sn_9_ fragments, and is structurally intermediate between [Ni_2_CdSn_18_]^6−^, where the fragments are clearly separated and [Pd_2_Sn_18_]^4−^, where they are completely fused. It therefore represents an intermediate stage in cluster growth. Analysis of the electronic structure suggests that the presence of the linear Fe–Fe–Fe unit is an important factor in directing reactions towards fusion of the two Sn_9_ units rather than the alternative of oligomerization *via* exo bond formation.

## Abstract 2

The effective oxidation state of the Sn_18_ unit determines the degree of fusion of the two Sn_9_ polyhedra in the series [Ni_2_CdSn_18_]^6−^, [Fe_3_Sn_18_]^4−^ and [Pd_2_Sn_18_]^4−^. The new [Fe_3_Sn_18_]^4−^ cluster represents an intermediate phase in the fusion process.

## Graphical abstract

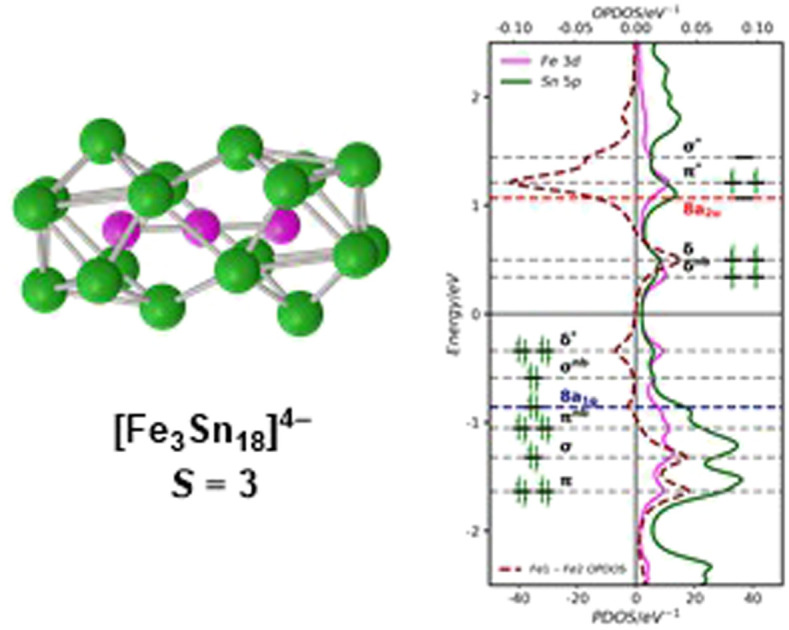

## Introduction

**Fig. 1:**
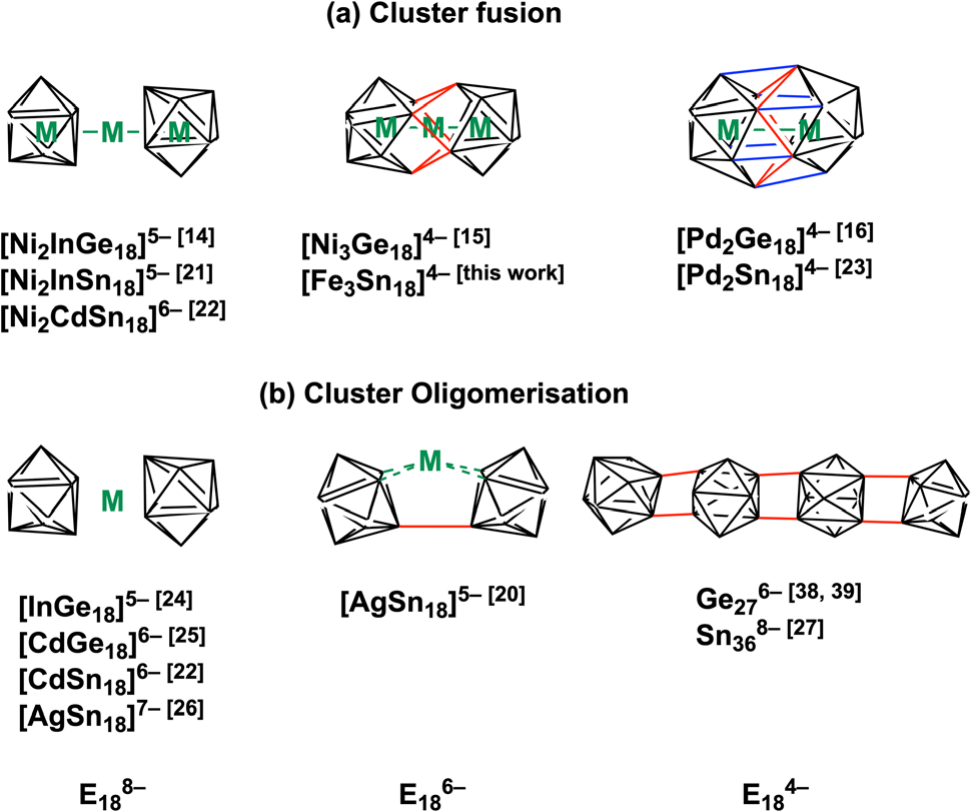
Cluster fusion *vs.* cluster oligomerisation of E_9_ polyhedra, E = Ge, Sn.^14–16,19–29^

The chemistry of Zintl ions, and in particular those containing endohedral metals, has been the subject of several recent reviews,^1–9^ and applications in catalysis and materials chemistry are beginning to emerge.^10,11^ The vast majority of these clusters are relatively small (14 main-group atoms or fewer) and contain a single transition metal ion, often with a closed-shell d^10^ configuration: classic examples include the icosahedral triad [Ni/Pd/PtPb_12_]^2−^,^12^ but the range of encapsulated metals now includes much of the d block. Larger clusters containing multiple transition metals are much less common but they offer the possibility of unusual magnetic phenomena and/or metal–metal bonding. Amongst the few known examples,^13^ the Ge_18_ series [Ni_2_InGe_18_]^5−^,^14^ [Ni_3_Ge_18_]^4−^,^15^ and [Pd_2_Ge_18_]^4−^ (ref. 16) (Fig. 1) maps out a progressive fusion of the two Ge_9_ polyhedra which are well separated in [Ni_2_InGe_18_]^5−^ but fully coalesced in the Pd cluster. [Ni_3_Ge_18_]^4−^ appears to be an intriguing intermediate case, where the fusion is only partially complete. It is far from clear how these clusters are actually formed *in situ*, but it is certainly plausible that the stepwise fusion of pre-formed polyhedral E_9_ or ME_9_ fragments is involved. Indeed, Sevov and Goicoechea proposed the fusion of NiGe_9_ and Ni_2_Ge_9_ units as a possible route to formation of [Ni_3_Ge_18_]^4−^,^17^ and Dehnen's analysis of fragmentation patterns for [TaGe_4_As_8_]^3−^ and [TaGe_6_As_6_]^3−^ identified cluster fragments such as [Ge_2_As_2_]^2−^ and [Ge_3_As]^3−^ that may play a role in growth.^18^ The challenge from a synthetic perspective is that these component polyhedra typically carry high negative charges, and so their close approach incurs a high coulombic penalty. Transition metal ions that can bridge two polyhedral units may, therefore, play an important role in fusion by buffering these repulsions and also, potentially, by removing excess electron density through the extrusion of metal in the elemental form. A further complication is that the oxidative fusion of clusters is, at least in principle, in competition with oxidative oligomerisation *via* the formation of *exo* E–E bonds (Fig. 1(b)). This phenomenon is well established in Ge chemistry where linked chains of Ge_9_ are known.^17,19^ A deeper understanding of the factors that control cluster growth and the balance between fusion and oligomerisation may provide access to a wider range of element combinations and compositions, and to tailored structural, magnetic and catalytic properties.

In this paper, we extend our recent work on the Zintl-ion chemistry of tin by reporting the synthesis of a new cluster, [Fe_3_Sn_18_]^4−^, which has a linear Fe_3_ chain and Fe–Fe bond lengths of 2.4300(9) Å. The Fe_3_ chain is of significant interest in its own right – there are few examples of metal–metal bonded units encapsulated inside Zintl clusters, and the short Fe–Fe distances are a clear *a priori* indication of strong bonding. Of equal interest is the structure of the Sn_18_ cage because the degree of fusion of the two Sn_9_ polyhedra appears to be mid-way between the completely separated limited, as observed in [Ni_2_CdSn_18_]^6−^, and the completely fused limit in [Pd_2_Sn_18_]^4^. [Fe_3_Sn_18_]^4−^ is, therefore, the Sn analogue of the Ge_18_ unit in [Ni_3_Ge_18_]^4−^. Our analysis of the electronic structure indicates that the E_18_ cages in [Ni_3_Ge_18_]^4−^ and [Fe_3_Sn_18_]^4−^ share a common −6 charge state, as does the Sn_18_ unit in [AgSn_18_]^5−^,^20^ where the two Sn_9_ units are not fused but rather oligomerised *via* an *exo* bond (Fig. 1(b)). A comparison of the different structural chemistry of these isoelectronic species offers a fascinating insight into the factors that control the balance between fusion in [Fe_3_Sn_18_]^4−^ and [Ni_3_Ge_18_]^4−^ and oligomerisation in [AgSn_18_]^5−^.

## Results and discussion

### Structure and properties of [Fe_3_Sn_18_]^4−^

**Fig. 2:**
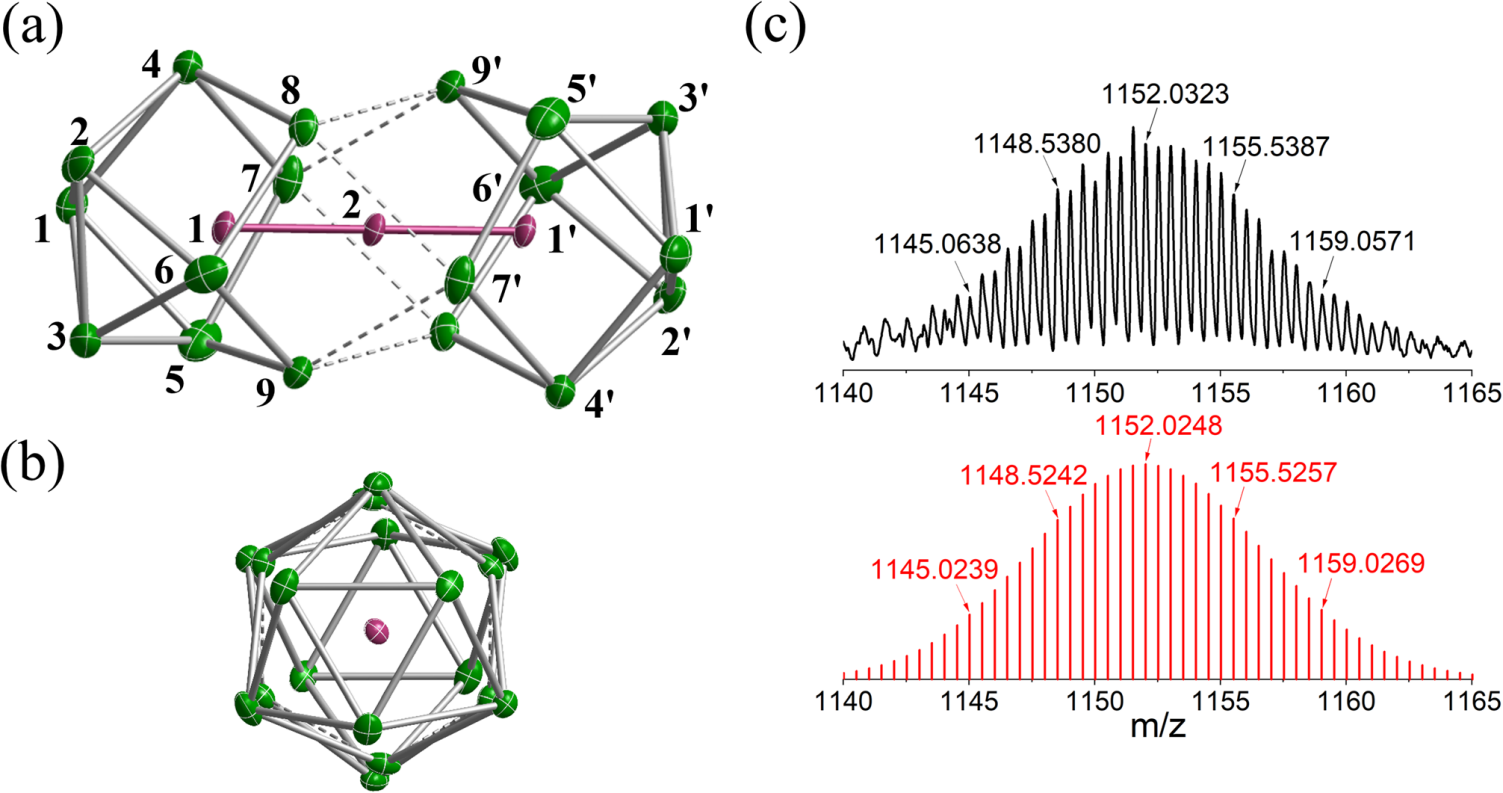
(a) and (b) Structure of anionic component, [Fe_3_Sn_18_]^4−^, of 1 and (c) the ESI-MS of a freshly-prepared solution of 1 in DMF.

**Fig. 3:**
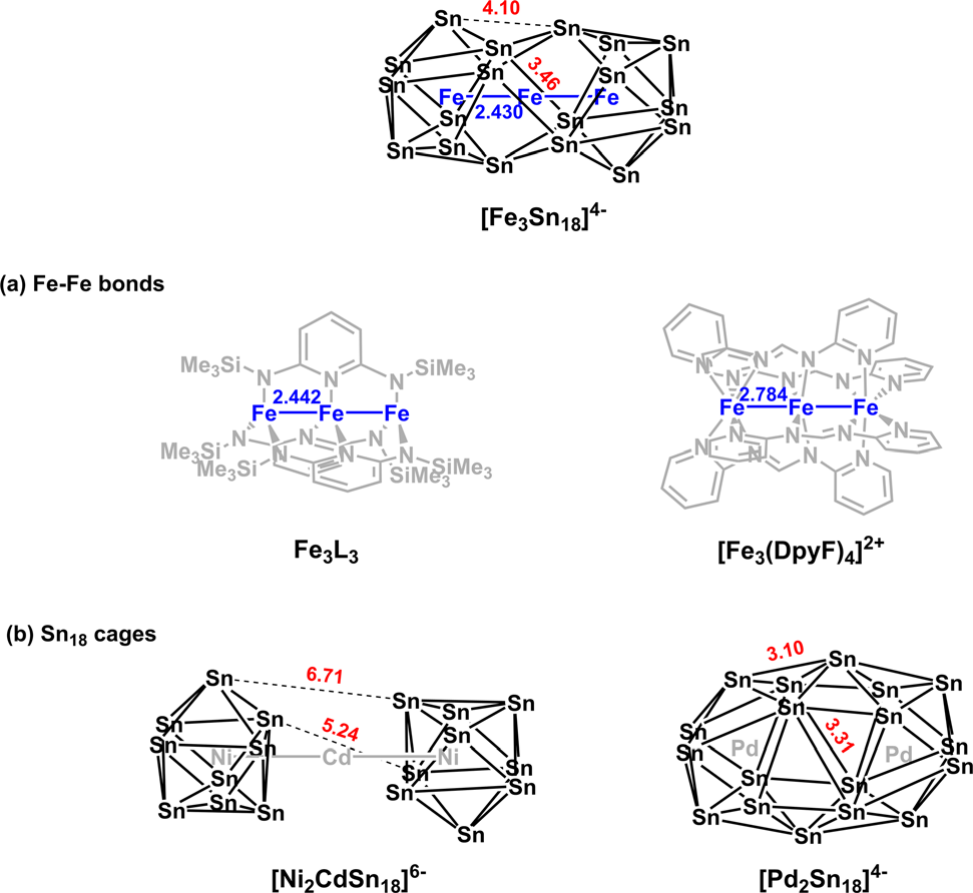
Structural comparisons with [Fe_3_Sn_18_]^3−^: (a) other linear Fe_3_ coordination compounds and (b) other E_18_ Zintl clusters.

The reaction of ethylenediamine (en) solutions of K_4_Sn_9_ with [K(thf)Fe(O^*t*^Bu)_3_]_2_ (thf = tetrahydrofuran) results in the formation of the tri-iron cluster [Fe_3_Sn_18_]^4−^ in the form of its [K(2.2.2-crypt)]^+^ salt [K(2.2.2-crypt)]_4_[Fe_3_Sn_18_] (1). Electrospray ionisation mass spectrometry (ESI-MS) of freshly-prepared DMF (DMF = dimethylformamide) solutions of 1 reveals a peak attributable to the dianion [Fe_3_Sn_18_]^2−^ (*m*/*z* 1152.0323 – note the peak-to-peak separations of 0.5 between isotopologues that confirm the −2 charge, Fig. 2(c)) and also a very weak signal assigned to the cation–dianion pair [K(2.2.2-crypt)Fe_3_Sn_18_]^−^ (*m*/*z* 2719.2300). It is common to observe only singly charged anions in the ESI-MS of Zintl clusters, but the large size of the Fe_3_Sn_18_ unit reduces the coulomb repulsion in the dianion to the extent that it is not ionized under the prevailing conditions. 1 crystallises in the monoclinic space group *P*2_1_/*c* and the unit cell contains a single anionic [Fe_3_Sn_18_]^4−^ cluster with four [K(2.2.2-crypt)]^+^ cations (Fig. 2(a) and (b), CCDC 2170116). The Sn_18_ unit adopts a *D*_3d_-symmetric structure based on two Sn_9_ polyhedra in a staggered, face-to-face arrangement, with a chain of three Fe centers aligned along the principal axis. In this section and the following discussion of the electronic structure, we focus first on the Fe_3_ chain, where Fe–Fe bonding is the primary interest, before turning to the Sn_18_ cage which we try to place in the wider context of Zintl-ion chemistry. The Fe–Fe bond lengths of 2.4300(9) Å in 1 are remarkably short, much shorter than those in the other known Fe_2_-containing Zintl cluster, [Fe_2_Ge_16_]^4−^ (2.636(3) Å). Even shorter bonds are known in Fe_2_ dimers such as the Fe^I^Fe^I^ paddlewheel complex^30^ (2.127 Å) and the (as-yet unknown) Fe_2_C_30_ (2.10 Å).^31^ Direct comparison with other Fe_3_ chains is restricted to classical coordination compounds such as Guillet's bis[(trimethylsilyl)amido]pyridine complex (Fig. 3(a), referred to henceforth as Fe_3_L_3_) where the Fe–Fe bond lengths are 2.4416(5) Å (ref. 32) and to the [Fe_3_(DpyF)_4_]^2+^ complex (DpyF = dipyridylformamide) first synthesised by Cotton and Murrillo^33^ and subsequently studied by Hillard and co-workers,^34^ where the Fe–Fe bond lengths are longer, at 2.7838(5) Å. These two Fe^II^Fe^II^Fe^II^ complexes share a common *S* = 6 ground state and a common formal σ bond order of 0.25 (per Fe–Fe bond), but differ in the distribution of electrons in the levels of π symmetry, with only Fe_3_L_3_ having an additional π component to the Fe–Fe bond. Correlations between bond order and bond length are notoriously difficult when bridging ligands are present, but nevertheless the similar bond lengths in [Fe_3_Sn_18_]^4−^ and Fe_3_L_3_ offers an initial indication that Fe–Fe π bonding may also be significant in the former. We return to this question in the following discussion of the electronic structure of the cluster. Turning our focus now to the structure of the Sn_18_ cage, we note first that the cluster can be viewed as two FeSn_9_ units, bridged by a third Fe center. We can make useful comparison to the pair of closely-related clusters identified in Fig. 3(b), [Ni_2_CdSn_18_]^6−^,^22^ and [Pd_2_Sn_18_]^4−^,^23^ where we judge the degree of fusion of the two Sn_9_ units in terms of two distinct Sn–Sn distances identified in Fig. 2, Sn8−Sn9′ and Sn4−Sn9′. The average values of these are 3.46 Å and 4.10 Å, respectively in [Fe_3_Sn_18_]^4−^ compared to 5.24 Å and 6.71 Å for [Ni_2_CdSn_18_]^6−^ and 3.31 Å and 3.10 Å for [Pd_2_Sn_18_]^6−^. The Sn8−Sn9′ bond lengths in [Fe_3_Sn_18_]^4−^ are therefore very similar to those in [Pd_2_Sn_18_]^4−^, but the Sn4−Sn9′ bonds are fully 1 Å longer. On this basis, we argue that the Sn_18_ cage in [Fe_3_Sn_18_]^4−^ represents an intermediate stage in the cluster fusion process that occupies the same central position in Sn cluster chemistry as [Ni_3_Ge_18_]^4−^ cluster^15^ does in the [Ni_2_InGe_18_]^5−^, [Ni_3_Ge_18_]^4−^, [Pd_2_Ge_18_]^4−^ series identified in Fig. 1.

### Electronic structure

**Fig. 4:**
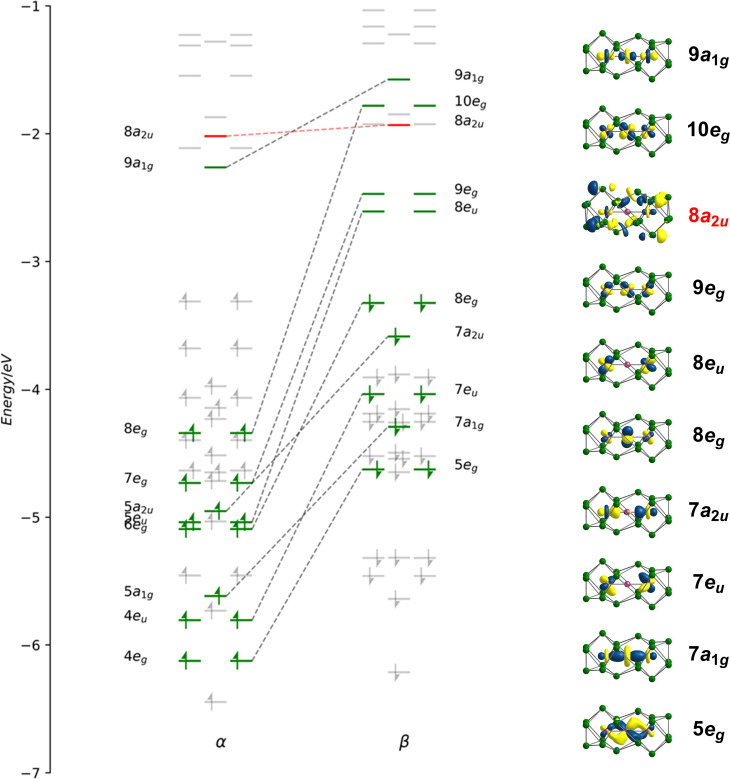
(a) Kohn–Sham orbitals for [Fe_3_Sn_18_]^4−^ in its ^7^A_2g_ ground state. Levels shown in green are the primarily Fe-based orbitals while the remainder, in grey, have dominant Sn character. Orbitals with similar spatial characteristics are joined by a dashed line.

Geometry optimisations using the PBE functional indicate that the lowest energy state for [Fe_3_Sn_18_]^4−^ is a spin septet (*S* = 3), ^7^A_2g_, with optimised Fe–Fe bond lengths of 2.45 Å, in excellent agreement with the available X-ray data (Table 1). The Fe–Sn and Sn–Sn bond lengths are also fully consistent with experiment. Despite multiple attempts, we have been unable to measure reproducible magnetic susceptibilities to confirm the paramagnetism of [Fe_3_Sn_18_]^4−^: this likely reflects the challenges in producing a homogeneous sample, and in avoiding oxidative degradation during the course of the experiment. The spin-polarised Kohn–Sham eigenvalues and eigenfunctions of the ^7^A_2g_ ground state are collected in Fig. 4: levels that are localised primarily on the Fe_3_ chain are shown in green while those localised primarily on Sn are in grey. The same data, in the form of projected density of states (PDOS) and overlap projected density of states (OPDOS), is presented and discussed in the ESI, Fig. S8.† Of the 100 valence electrons of the cluster, we can identify 22, colored green, that are distributed over the 15 linear combinations of Fe 3d orbitals in Fig. 4 (4e_g_, 5a_1g_, 4e_u_, 7a_2u_, 6e_g_, 5e_u_, 7e_g_, 8e_g_ and 9a_1g_ in the α set). It is notoriously difficult to assign oxidation formal states in endohedral Zintl clusters, where transition- and main-group metal orbitals are typically well mixed, but the presence of 22 valence electrons indicates a Fe^2^_3_^+^ chain, and hence a Sn_18_ cluster in a −6 oxidation state.

**Table 1:** Selected bond lengths from crystallographic and DFT-optimised structures for the [M_3_E_18_]^4−^ family (all distances in Å). See Fig. 2(a) for atom numbering

		M1–M2	M1–E1	M2–E8	E2–E8	E2–E4	E6–E9	E8−E9′	E4−E9′	Ref.
[Fe_3_Sn_18_]^4−^	X-ray (100 K)	2.4300(9)	2.882	2.689	3.690	3.001	3.004	3.460	4.10	This work
	DFT (^7^A_2g_)	2.45	2.98	2.70	3.64	3.07	3.04	3.50	4.22	
[Ni_3_Ge_18_]^3−^	X-ray (100 K)	2.395(1)	2.487	2.529	3.016	2.749	2.612	3.47	4.05	15
	DFT (^1^A_1g_)	2.43	2.50	2.52	3.09	2.76	2.66	3.29	4.01	
[Ni_2_CdSn_18_]^6−^	X-ray (100 K)	4.201	2.595	3.206	3.549	3.011	2.981	5.24	6.71	22
	DFT (^1^A_1g_)	4.26	2.64	3.13	3.71	3.07	3.02	5.32	6.81	

Of the 15 metal-based orbitals, only one, the strongly Fe–Fe–Fe σ anti-bonding 9a_1g_ orbital, is vacant in both spin-α and spin-β manifolds, while the complementary σ bonding and non-bonding orbitals, 5a_1g_α, 7a_1g_β and 5a_2u_α, 7a_2u_β, are doubly occupied: the σ^2^σ^*n*b2^σ*^0^ configuration gives a net σ bond order of 0.5 per Fe–Fe bond. Fe–Fe π and δ interactions are mixed in the orbitals of e_g_ and e_u_ symmetry, but the π interactions are primarily contained in 4e_g_, 4e_u_ and 8e_g_ in the α manifold, 5e_g_, 7e_u_ and 10e_g_ in β. The prominent positive and negative peaks in the OPDOS shown in ESI, Fig. S8,† corresponding to 5e_g_β (π bonding) and 10e_g_β (π antibonding), respectively, confirm the very significant π overlap. The π^4^π^*n*b4^π*^2^ configuration then defines a formal Fe–Fe π bond order of 0.5 per bond. There are no large peaks in the OPDOS for the orbitals with dominant Fe–Fe δ symmetry (5e_u_α, 7e_g_α, 9e_g_α, 8e_u_β, 8e_g_β, 9e_g_β), so δ bonding can be assumed to be negligible, as might be expected at a distance of 2.4300(9) Å. The overall formal Fe–Fe bond order is therefore 1.0 per Fe–Fe bond made up of 
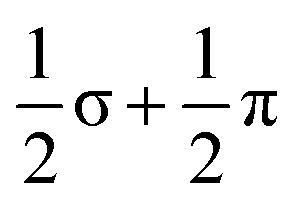
. Returning to the comparison with the coordination complexes [Fe(DpyF)_4_]^2+^ (ref. 34) and Fe_3_L_3_,^32^ identified in Fig. 3, the overall oxidation state of the Fe_3_ unit is lower in [Fe_3_Sn_18_]^4−^ (Fe^2^_3_^+^*vs.* Fe^6^_3_^+^) and the Fe–Fe σ* orbital is unoccupied, both of which contribute to the lower multiplicity (*S* = 3 *vs. S* = 6) and stronger Fe–Fe bonding in the cluster compared to the coordination complexes.

To place the Fe–Fe bonding into the wider context of Zintl cluster chemistry, we can make a connection to Sevov and Goicoechea's [Ni_3_Ge_18_]^4−^, which is a spin singlet with a total valence electron count of 106. The additional six electrons occupy the three doubly degenerate, metal-based, spin-β orbitals, 8e_u_β, 9e_g_β and 10e_g_β, eliminating the π component of the metal–metal bond but leaving the σ^2^σ^*n*b2^σ*^0^ framework intact. The M–M Mayer bond order^35^ is reduced from 0.95 in [Fe_3_Sn_18_]^4−^ to 0.45 in [Ni_3_Ge_18_]^4−^, and the delocalisation index (DI)^36^ from 0.69 to 0.44, both metrics pointing to a significant π component to the Fe–Fe bond in [Fe_3_Sn_18_]^4−^. The addition of 6 electrons to the metal chain generates a Ni^2^_3_^+^ unit and, hence, a Ge_18_ cluster in the −6 oxidation level. Switching our focus now to the Sn_18_ cage, we can identify a single vacant orbital, 8a_2u_, picked out in red in Fig. 4, that has Sn–Sn σ* character between the Sn_3_ faces bound to the central Fe atom (Sn8−Sn9′ in Fig. 2). This orbital, along with its doubly-occupied Sn–Sn bonding counterpart, generates a 6-center-2-electron bond that links the two Sn_9_ units.

### Cluster fusion *vs.* cluster oligomerisation

In the previous section we have established a link between the new cluster [Fe_3_Sn_18_]^4−^ and [Ni_3_Ge_18_]^4−^ through their common oxidation level of −6 for the E_18_ cluster unit. In this section, we try to identify broader relationships between the family of clusters with 18 tetrel vertices (E_18_) but rather different structures. Amongst these, we can pick out the two pairs, [Ni_2_CdSn_18_]^6−^ and [Ni_2_InGe_18_]^5−^and [Pd_2_Sn_18_]^4−^ and [Pd_2_Ge_18_]^4−^ shown in Fig. 1 all of which share a common point symmetry, *D*_3d_, with [Fe_3_Sn_18_]^4−^ and [Ni_3_Ge_18_]^4−^. The oxidation states of the E_18_ unit in these four clusters are rather easier to establish: in [Pd_2_Ge_18_]^4−^ and [Pd_2_Sn_18_]^4−^, the Pd atoms are diamagnetic and can be assigned straightforwardly a formal oxidation state of 0 (d^10^), leaving the Sn_18_ unit in a −4 charge state. In [Ni_2_InGe_18_]^5−^ and [Ni_2_CdSn_18_]^6−^, in contrast, the diamagnetism implies Ni^0^, Cd^2+^ and In^3+^ (all d^10^), defining a charge of −8 on the Sn_18_ unit. Structurally, the two E_9_ units are separated by more than 4.0 Å in [Ni_2_InGe_18_]^5−^ and [Ni_2_CdSn_18_]^6−^ but are very tightly compressed in [Pd_2_Ge_18_]^4−^ and [Pd_2_Sn_18_]^4−^, where the E_18_ unit forms a continuous ellipsoidal cage. From a structural perspective, [Fe_3_Sn_18_]^4−^ and [Ni_3_Ge_18_]^4−^ appear to be precisely intermediate between the two limits, with two partially but not fully coalesced E_9_ units, consistent with the formal charge assignment of Sn_18_^6−^.

**Fig. 5:**
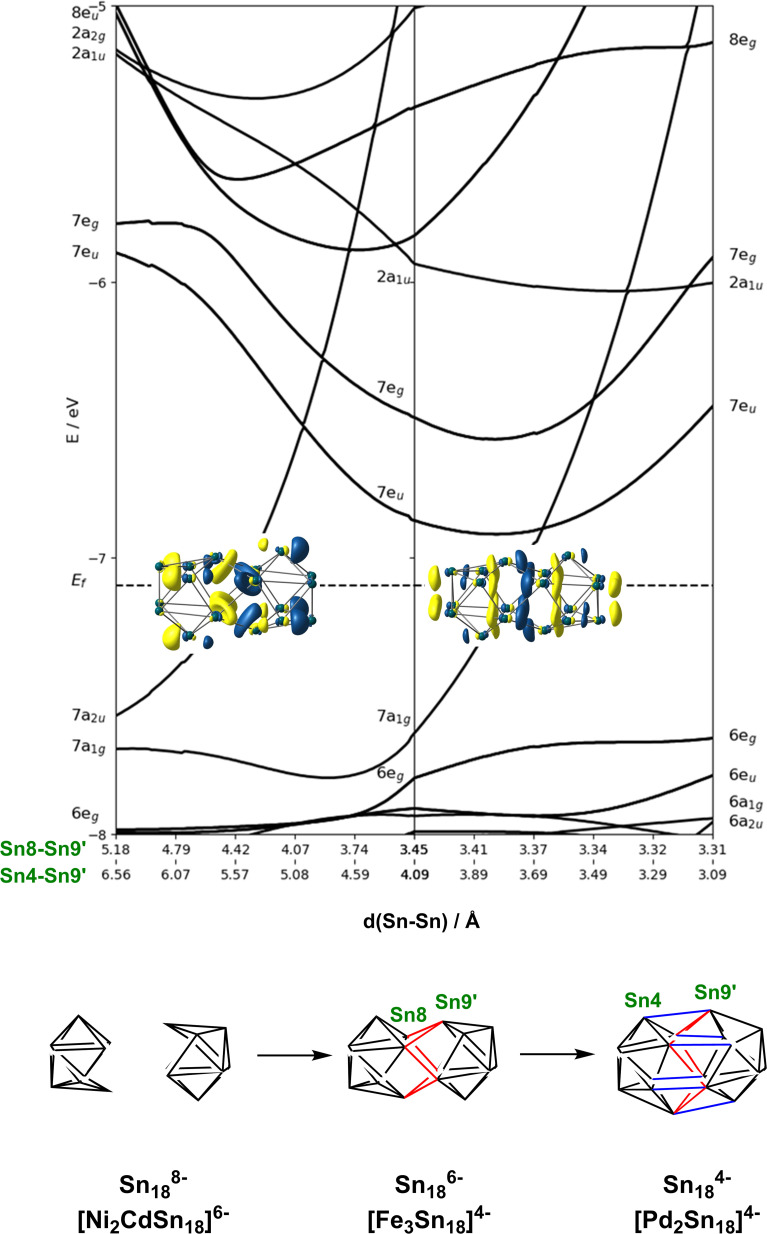
Walsh diagram showing the coalescence of the two Sn_9_ polyhedra to a single ellipsoidal Sn_18_ unit. The figure is generated by interpolating between the optimised structures of the Sn_18_ unit as it is found in the optimised geometries of [Ni_2_CdSn_18_]^6−^, [Fe_3_Sn_18_]^4−^ and [Pd_2_Sn_18_]^4−^.

If we wish to analyse the electronic origins of these structural trends we are faced with the immediate problem that in some cases the clusters contain 3 transition metal ions but in others only 2. In order to circumvent this difficulty, we choose to focus on the electronic structure of the empty cage, Sn_18_, and explore its dependence on charge state: −8 → −6 → −4. The relationship between structure and charge state can be made explicit by the Walsh diagram for the isolated E_18_ cluster shown in Fig. 5 (calculated using extended Hückel theory). This figure is constructed by extracting the structures of the Sn_18_ units from DFT optimisations of [Ni_2_CdSn_18_]^6−^, [Fe_3_Sn_18_]^4−^ and [Pd_2_Sn_18_]^4−^ and interpolating between these three geometries. A comment on the choice of reaction coordinate is necessary here. The fusion of the two Sn_9_ units proves to be highly asynchronous: the structural impact of the first 2-electron oxidation is very different from the second 2-electron oxidation. In such circumstances, no single structural parameter can adequately capture the changes occurring across the entire spectrum, from 2 × Sn_9_^4−^ on the left to Sn_18_^4−^ on the right. We therefore choose to identify two distinct Sn–Sn distances that serve as independent measures of structural change. The Sn8−Sn9′ distance is closely related to the distance between the centroids of the two Sn_9_ units, and it varies rapidly as we go from 2 × Sn_18_^4−^ to Sn_18_^6−^, and then more slowly from Sn_18_^6−^ to Sn_18_^4−^. The Sn8−Sn9′ distance, in contrast, varies strongly in the left half of the diagram, but is relatively constant as we move from Sn_18_^6−^ to Sn_18_^4−^. We can, therefore associate the first 2-electron oxidation with a relative motion of the two Sn_9_ units towards each other, such that both Sn4−Sn9′ and Sn8−Sn9′ contract. The second 2-electron step is then associated almost exclusively with the formation of the Sn4−Sn9′ bonds, with little further change in Sn8−Sn9′.

At the separated limit (left hand side of Fig. 5) there is a total of 40 low-lying valence orbitals (up to 7a_2u_) that can accommodate 80 valence electrons, the count for Sn_18_^8−^. The transition from this separated limit to the intermediate structure typical of [Fe_3_Sn_18_]^4−^ (or [Ni_3_Ge_18_]^4−^) involves a reduction in the separation between the centroids of the two Sn_9_ units, resulting in contraction of both the Sn8−Sn9′ and Sn4−Sn9′ distances. The result is the rapid destabilisation of a single orbital, 7a_2u_, that is antibonding across Sn8−Sn9′ – this is the Sn–Sn antibonding orbital discussed previously in the context of Fig. 4 (where it was labelled 8a_2u_ due to the presence of a lower-lying Fe/Ni-based level of the same symmetry that is obviously absent in the empty cluster). In the second step, from the intermediate structure found in [Fe_3_Sn_18_]^4−^ to the fully coalesced one in [Pd_2_Sn_18_]^4−^, the Sn4−Sn9′ distance contracts from 4.22 Å to 3.10 Å, causing a rapid destabilisation of a second cluster-based orbital, 7a_1g_, which is bonding with respect to the Sn8−Sn9′ contact but strongly anti-bonding with respect to Sn8−Sn9′. We note here that Lin and co-workers have also analysed the fusion of two PdSn_9_ units from the perspective of the ‘principal interacting orbital’ model,^37–39^ where they identified a σ-symmetry interaction between ‘principal interacting orbitals' localised on the Sn4 and Sn9′ atoms. To the extent that the structurally characterised clusters illustrated in Fig. 1 can be viewed as snapshots of the oxidative coalescence of two separated Sn_9_ clusters, it seems that the 4-electron oxidation of Sn_18_^8−^ to Sn_18_^4−^ is a rather asynchronous one, with the two units coming together first *via* the formation of Sn8−Sn9′ bonds (shown in red in Fig. 5), followed by a distinct second 2-electron oxidation step that leads to formation of the Sn4−Sn9′ bonds (shown in blue in Fig. 5), completing the fusion of the two units.§§[Fe_3_Sn_18_]^2−^ was identified as a prominent peak in the ESI-MS in Fig. 2. This cluster is formed by 2-electron oxidation of the [Fe_3_Sn_18_]^4−^ and so, if the oxidation is Sn-based, the cage is valence isoelectronic with that in [Pd_2_Sn_18_]^4−^. However, the optimised structure does not show a completely coalesced cage, but rather one that is similar to [Fe_3_Sn_18_]^4^− despite the 2-electron oxidation. The presence of the Fe_3_ unit clearly prevents the close approach of the two Sn_9_ units that is possible for [Pd_2_Sn_18_]^4^−.

In the introduction we noted that there is, in principle, a competing pathway for oxidative coupling of Zintl clusters that leads to oligomerisation *via exo* bond formation rather than fusion to form a single ellipsoidal cage. This is precisely what is observed in the oxidation of [AgSn_18_]^7−^ (ref. 26) to [AgSn_18_]^5−^ (Fig. 1) where the charge on the Sn_18_ unit (assuming a redox non-innocent Ag^+^ ion) is reduced from −8 to −6, precisely the same as in the [Ni_2_CdSn_18_]^6−^ to [Fe_3_Sn_18_]^4−^ comparison. What, then, are the factors that determine the preference for fusion of the two Sn_9_ units in [Fe_3_Sn_18_]^4−^ with retention of three-fold rotational symmetry but oligomerisation in [AgSn_18_]^5−^? From an electronic perspective, the Sn_18_ units in the two clusters both have a 2-electron bond linking the two Sn_9_ units – the only difference is that in [Fe_3_Sn_18_]^4−^ this bond is delocalised over 6 Sn–Sn contacts, each with a formal bond order of 
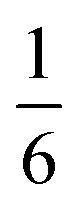
, while in [AgSn_18_]^5−^ it is localised on one. It is possible that the preference for a fused architecture with a 6-center-2-electron bond in [Fe_3_Sn_18_]^4−^ (and also in [Ni_3_Ge_18_]^4−^) is connected to the presence of the underlying Fe–Fe–Fe or Ni–Ni–Ni bonded framework, which provides a rigid ‘strut’ that resists the bending at the central metal necessary to form a localised *exo* Sn–Sn bond. Where metal–metal bonding is absent, as it necessarily is in [AgSn_18_]^5−^, bending to form a localised 2-center-2-electron bond is the preferred outcome: a series of DFT calculations on different isomers of [AgSn_18_]^5−^ confirms a 0.2 eV preference for the bent structure shown in Fig. 1 over the alternative *D*_3d_-symmetric [Fe_3_Sn_18_]^4−^-like alternative. Taking the argument a step further, a second 2-electron oxidation step could, in principle, generate clusters with two *exo* bonds linking the Sn_9_ units as an alternative to forming the coalesced cage typical of [Pd_2_Sn_18_]^4−^ – doubly bonded E_9_ units of this kind have been identified in the Ge_27_^38,39^ and Sn_36_ nanorod^27^ where again there is no underlying metal–metal bonded framework to oppose the bending.

## Summary and conclusions

In this paper, we have reported the synthesis and structure of a new Zintl-ion cluster, [Fe_3_Sn_18_]^4−^, containing a linear Fe_3_ chain with short Fe–Fe bond lengths of 2.4300(9) Å. Electronic structure analysis indicates the presence of both Fe–Fe σ and π bonding, with a formal net bond order of 1.0 (
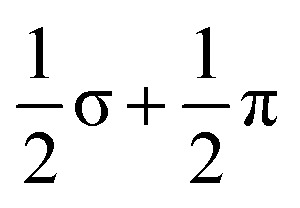
) per Fe–Fe bond. The cluster is structurally similar to the [Ni_3_Ge_18_]^4−^ anion reported previously by Sevov and co-workers, although the Ni–Ni bonding in that case lacks the π component. The structure of the Sn_18_ unit in [Fe_3_Sn_18_]^4−^ is intermediate between that in [Ni_2_CdSn_18_]^6−^, where the two Sn_18_ units are almost completely separated, and [Pd_2_Sn_18_]^4−^, where they are completely fused to form a continuous ellipsoidal Sn_18_ unit. These structural differences correlate with the redox level of the cage, with successive 2-electron oxidations starting from Sn_18_^8−^ leading first to partial fusion of the two cages and then to their complete coalescence. One of the obvious challenges in forming ever larger Zintl ions from smaller fragments is that the latter carry high negative charges, so their close approach necessarily involves a substantial coulomb barrier. The identification of [Fe_3_Sn_18_]^4−^ as an intermediate stage of cluster fusion presents the intriguing possibility that the central metal cation may act as a buffer, templating the close approach of the anionic components. Transfer of electron density from the main-group cage to the transition metal may then drive the fusion of the two polyhedral fragments, with concomitant reduction of the cations and their expulsion as metal atoms, as is observed, for example, in NaSi, where elevated pressures lead to the formation of Na metal with concomitant amorphisation of the Si_4_ cluster units through Si–Si bond formation.^40^ The presence of Fe–Fe bonding appears to play an important role in this process by preventing bending at the central metal atom, directing the reaction towards cluster fusion rather than the competing oxidative oligomerisation observed in the [AgSn_18_]^7−/5−^ pair.

## Experimental section

### Materials and reagents

All manipulations and reactions were performed under a nitrogen atmosphere using standard Schlenk or glovebox techniques. Ethylenediamine (en) (Aldrich, 99%) and DMF (Aldrich, 99.8%) were freshly distilled by CaH_2_ prior to use, and stored in N_2_ prior to use. Tol (Aldrich, 99.8%) was distilled from sodium/benzophenone under nitrogen and stored under nitrogen. 2.2.2-crypt (4,7,13,16,21,24-hexaoxa-1,10-diazabicyclo (8.8.8) hexacosane, purchased from Sigma-Aldrich, 98%) was dried in vacuum for one day prior to use. K_4_Sn_9_ was synthesised by heating a stoichiometric mixture of the elements (K: +99% and Sn: 99.99% all from Aladdin) at 850 °C for 36 h in a niobium tube. [K(thf)Fe(O^*t*^Bu)_3_]_2_ was prepared according to literature methodology.^41^

### Synthesis

#### [K(2.2.2-crypt)]_4_[Fe_3_Sn_18_] (1)

In a 10 mL vial, K_4_Sn_9_ (122 mg, 0.100 mmol) and 2.2.2-crypt (113 mg, 0.3 mmol) were dissolved in en (*ca.* 3 mL) and stirred for 30 min, resulting a dark brown solution. Then [K(thf)Fe(O^*t*^Bu)_3_]_2_ (33 mg, 0.043 mmol) was dispersed in toluene (0.5 mL), producing a light pink suspension, and then added dropwise to the above mixture. After stirring for 3 hours at room temperature, the resulting brown solution was filtered through glass wool and transferred to a test tube, then carefully layered by toluene (*ca.* 3 mL) to allow for crystallisation. Small brown block-like crystals of 1 (10% yield based on the K_4_Sn_9_ precursor) were isolated after two weeks.

### X-ray crystallography

Crystallographic data for 1 were collected on Rigaku XtalAB Pro MM007 DW diffractometer with graphite monochromated Cu Kα radiation (*λ* = 1.54184 Å). The crystal structure was solved using direct methods and then refined using SHELXL-2014 (ref. 42) and Olex2,^43^ with all non-hydrogen atoms refined anisotropically during the final cycles. All hydrogen atoms of the organic molecule were placed by geometrical considerations and were added to the structure factor calculation. The SQUEEZE procedure^44^ to remove the solvent molecules which could not be modeled properly. A summary of the crystallographic data for the title compounds is presented in the ESI, Tables S1 and S2.† These data can be obtained free of charge from The Cambridge Crystallographic Data Centre.

### Computational details

All calculations are performed using density functional theory as implemented in the ADF 2021.104 package.^45^ The Perdew–Burke–Ernzerhof (PBE) functional^46^ was used and triple-zeta basis sets included with two polarization functions are used for all atoms.^47^ All electrons are treated as valence in the calculations. Relativistic effects were incorporated using the Zeroth-Order Regular Approximation (ZORA).^48^ A Conductor-like Screening Model (COSMO) with dielectric constant of 78.39 was used to simulate the confining environment of the ionic lattice.^49^ A fine numerical grid was used for the integrations (grid setting ‘verygood’),^50^ and the calculations were considered converged when the commutator of the Fock and density matrices was below 10^−6^. Optimized structures were confirmed to be minima through the absence of imaginary frequencies.^51^ The sensitivity of the results to choice of functional was also explored by repeating the calculations using the M06-L and PBE0 functionals.^52,53^ The Walsh diagram in Fig. 5 was calculated using Extended Hückel theory with the following parameters for the 5s and 5p valence orbitals of Sn: 5s *H*_ii_ = −16.16 eV, *ζ* = 2.30, 5p *H*_ii_ = −7.32 eV, *ζ* = 2.00. The reaction coordinate was defined by interpolating between the structures of the Sn_18_ unit as it is found in the optimised structures of [Ni_2_CdSn_18_]^6−^, [Fe_3_Sn_18_]^4−^ and [Pd_2_Sn_18_]^4−^. A python script to perform these calculations is available on request.

## Data availability

Crystallographic data are available from the CCDC. Full details of optimised geometries are summarised in the ESI.† Input files are available from the authors on request.

## Author contributions

W.-X. C. and C.-C. S. performed the synthesis and characterisation, Z. L. and H. W. T. M. performed the computational analysis. Z.-M. S. and J. E. M. conceived the project and supervised the experimental and computational aspects of the research, respectively. All authors contributed to the preparation of the manuscript.

## Conflicts of interest

There are no conflicts to declare.

## Supplementary Material

SC-015-D3SC04709A-s001
